# Design preferences for global scale: a mixed-methods study of “glocalization” of an animated, video-based health communication intervention

**DOI:** 10.1186/s12889-021-11043-w

**Published:** 2021-06-25

**Authors:** Maya Adam, Rachel P. Chase, Shannon A. McMahon, Kira-Leigh Kuhnert, Jamie Johnston, Victoria Ward, Charles Prober, Till Bärnighausen

**Affiliations:** 1 Department of Pediatrics, Stanford University, Stanford, California, USA; 2 Independent Researcher, Columbus, Ohio, USA; 3 Heidelberg Institute of Global Health at the Faculty of Medicine, Heidelberg University, Heidelberg, Germany; 4 Johns Hopkins Bloomberg School of Public Health, Baltimore, Maryland, USA; 5 Digital Medic South Africa, Stanford University Center for Health Education, Stanford, California, USA; 6 Stanford University Center for Health Education, Stanford, California, USA; 7 Harvard Center for Population and Development Studies, Harvard T.H. Chan School of Public Health, Cambridge, Massachusetts, USA; 8 Wellcome Trust’s Africa Health Research Institute (AHRI), KwaZulu-Natal, South Africa

**Keywords:** Global health, Health communication, Health literacy, Health education, Human-centered design, Community health promotion

## Abstract

**Background:**

Designing health communication interventions for global scaling promotes health literacy and facilitates rapid global health messaging. Limited literature explores preferences for animation prototypes and other content characteristics across participants in different global regions. Prior research underscores an urgent need for health communication interventions that are compelling and accessible across culturally and geographically diverse audiences. This study presents feedback from global learners on animation design preferences and other key considerations for the development of educational video content intended for global adaptation and scaling.

**Methods:**

We used a mixed-methods, sequential explanatory design, with a qualitative descriptive approach to the analysis of the qualitative data. We recruited participants from an international group of learners enrolled in a massive open online course. Through an online quantitative survey (*n* = 330), we sought preferences from participants in 73 countries for animation design prototypes to be used in video-based health communication interventions. To learn more about these preferences, we conducted in-depth interviews (*n* = 20) with participants selected using maximum variation purposive sampling.

**Results:**

Generally, respondents were willing to accept animation prototypes that were free of cultural and ethnic identifiers and believed these to be preferable for globally scalable health communication videos. Diverse representations of age, gender roles, and family structure were also preferred and felt to support inclusive messaging across cultures and global regions. Familiar-sounding voiceovers using local languages, dialects, and accents were preferred for enhancing local resonance. Across global regions, narratives were highlighted as a compelling approach to facilitating engagement and participants preferred short videos with no more than two or three health messages.

**Conclusions:**

Our findings suggest that global learners may be willing to accept simplified visuals, designed for broad cross-cultural acceptability, especially if the content is localized in other ways, such as through the use of locally resonating narratives and voiceovers. Diverse, inclusive portrayals of age, gender roles and family structure were preferred.

**Supplementary Information:**

The online version contains supplementary material available at 10.1186/s12889-021-11043-w.

## Background

Effective and accessible health communication media interventions are increasingly recognized as key strategies for increasing basic health literacy and promoting healthy behaviors [1–5]. Researchers have thus called on professionals in the related fields of health communication, health education and health literacy to join forces in developing interventions that are compelling and effective for promoting behavior change [6]. A growing body of research also emphasizes the need to design communication interventions that are accessible across diverse global audiences [1, 3, 6, 7]. Key solutions could lie in designing interventions for “glocalization”, the adaptation of global content for local resonance [8].

Research suggests that health communication for global health literacy promotion needs to take into account cultural factors that may influence health in different target audiences. Thoughtful integration of these determinants has been identified as a cornerstone of effective interventions [3, 9, 10]. If our goal is to promote health literacy globally, including reaching marginalized audiences, we must design health communication interventions from the outset with an appreciation for global cultural variation [9, 11, 12].

Socio-cultural considerations influencing message design, narrative, and visual elements require close attention when designing animated health communication interventions [13, 14]. Prior research on the use of short, animated videos to promote agricultural knowledge gains in low- and middle-income countries, showed high long-term retention of information (98%) and solution adoption (89%) [15]. This research underscores the potential of short, animated videos [16] dubbed in local languages, to cost-effectively reach a broad range of global learners, including those facing literacy barriers and geographic isolation [17].

### The pilot intervention in South Africa

In 2018, we created a video-based maternal child health (MCH) communication intervention in collaboration with the South African National Department of Health. The intervention was aligned with and intended to amplify their existing, text based, national MCH education program, called The Road to Health. Our shared goal was to design and produce a series of educational videos, intended to optimally engage a broad spectrum of mothers, fathers and caregivers across this culturally diverse country [14]. In designing this intervention, we aimed to overcome four key challenges using existing health communication approaches and digital solutions:

1. In order to develop animation prototypes that were acceptable and compelling to culturally diverse mothers, fathers and caregivers across South Africa, we intentionally avoided cultural identifiers such as hairstyles, facial features and traditional clothing, (see Additional file 1). This approach is informed by the theory of Universal Design for Learning, a theoretical framework that has been successfully applied to the design of broadly acceptable, accessible and culturally inclusive health literacy interventions [3].
2. To overcome high data costs, we designed vector-based, two-dimensional animations compatible with small file sizes (2-10 MB) and optimized for mobile viewing.
3. To overcome literacy barriers, facilitate engagement and easy translation into different languages, we used a narrative, entertainment-education approach. This approach has demonstrated promise in communicating health messages, persuading and motivating behavior change [4].
4. To facilitate future “glocalization”, (ie: localizing content created for a broad, global audience), we applied lessons learned from the children’s entertainment-education industry. Research documenting the glocalization of Sesame Street by the Children’s Television Network, in the 1970’s, demonstrated the power of a structured approach to local language dubbing [18, 19].

**Additional file 1.** Kangaroo Mother Care (a short, animated video demonstrating the vector-based approach to character development free of cultural identifiers).

A major goal during the design of the pilot intervention, was to enhance emotional appeal and character identification, highlighted as important strategies in the behavior change, communication science, and education literature [18–23]. Following the launch of the intervention [24], we received requests to adapt it for use in other global regions. This raised the important question of whether or not the design prototypes would resonate across global audiences.

There is a shortage of research on visual design preferences for health communication across global audiences. In particular, mixed-methods approaches would help to provide both a broad sense of global design preferences, as well as a more nuanced understanding of those preferences. In this study, we summarize feedback from global learners on a set of animation design prototypes developed for diverse global audiences.

## Methods

### Mixed methods approach

In this study, we used a mixed-methods, sequential explanatory study design with a qualitative descriptive approach [25, 26]. The study consisted of a quantitative survey of preferences, followed by qualitative in-depth interviews. We used the results of the quantitative survey to a) inform the selection of interview participants and b) develop the interview guide for the second phase of the study (see Additional file 2).

### Quantitative survey of preferences

**Fig. 1:**
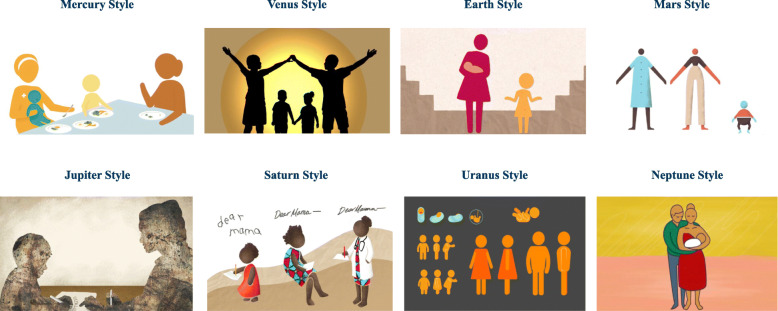
Design prototypes for animated health communication videos. * The earliest formative feedback, shaping the development of the eight prototypes, was gathered in South Africa in 2018, through interactions with South African National Department of Health and a large community-based maternal child health organization engaged in community health promotion. ** Prototypes were named after planets to facilitate discussing them without appearing to rank the prototypes or highlighting any specific feature of the prototype

We applied a Human Centered Design (HCD) approach to develop eight animation prototypes (see Fig. 1) [14]. HCD involves a process of rapid prototyping, iterative cycles of feedback from target audiences and re-design by the development team [27]. The earliest formative feedback, shaping the development of the prototypes [14], was gathered in South Africa in 2018, through our interactions with the South African National Department of Health and a large community-based maternal child health organization engaged in community health promotion. The number of prototypes and their primary health messages were pragmatic decisions based upon the availability of resources, including the expertise of four artists who designed the prototypes according to our stated goals, as well as the real-world health messaging needs of our collaborating health organizations.

We recruited participants for the quantitative survey of preferences from a cohort of learners enrolled in a global, massive open online course, Stanford Introduction to Food and Health [28]. The first author is lead instructor for this course, offered on Coursera, a massive open online learning platform serving more that 35 million learners worldwide. Learners enrolled in the Stanford Food and Health course represent more than 95 countries and enroll in cohorts, with a new cohort beginning every 2 weeks. In April 2019, we gauged interest in participating in a research study, among the most recent cohort of course completers. All learners who expressed interest in participating in research on health communication (*n* = 642) were invited to complete the preferences survey, where the 8 design prototypes were presented (see Fig. 1). We obtained informed consent digitally via an information sheet, sent to learners by email. Participants were not offered any financial or other incentives for their involvement. Three hundred and thirty participants, from 73 countries, completed the quantitative survey of preferences. We grouped participants by 9 global regions, using the World Health Organization’s definition of 6 global regions and sub-dividing three of these regions (the Americas, Europe and the Western Pacific region) to help further contextualize participants’ responses. The demographic characteristics of our participants are described in Table 1.

**Table 1:**
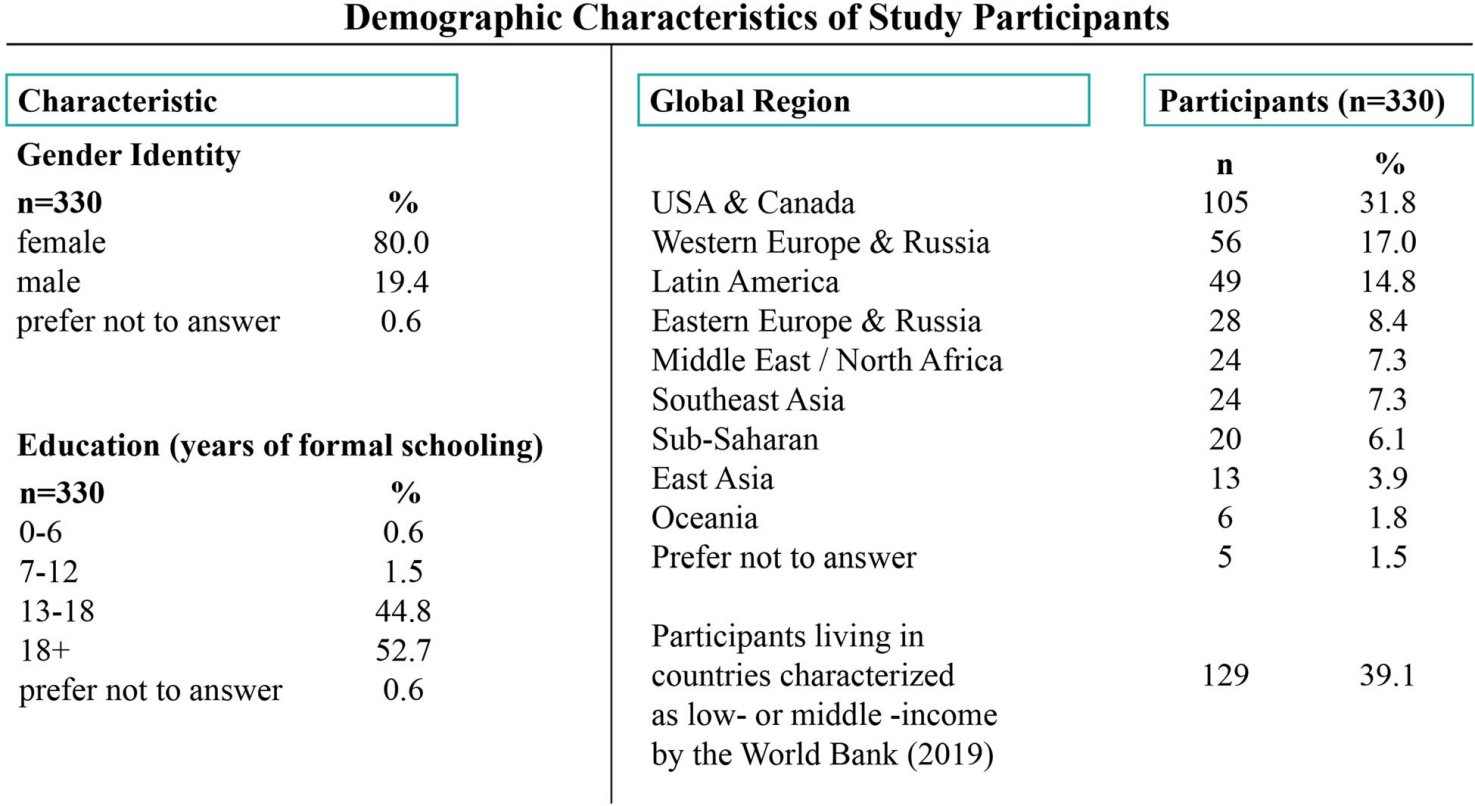
Demographic characteristics of study participants

Respondents indicated preferences for the animation prototypes in several ways. For each prototype, participants were asked to express whether or not they expected the prototype to resonate with people in their country as well as ranking the prototypes according to their own preferences. Participants were then invited to sort prototypes into categories of would, might, or would not resonate with a global audience and finally, participants rated all prototypes on a 6-point scale.

From the survey responses, we calculated the percentage of respondents characterizing each prototype as “would resonate within my country” as well as the percentage of respondents that ranked each prototype as a) their preferred prototype or b) within their top two preferred prototypes. We then analyzed the survey responses by global region, examining which prototypes were characterized as first, second or third choices for both global and local resonance. Finally, we calculated the average ratings of each prototype on a 6-point scale.

Survey respondents were also invited to share unstructured feedback on each prototype, using an open-feedback comment box embedded within the survey. This qualitative data, collected as part of the quantitative survey, was then analyzed by the research team through open coding. Codes that emerged from the data were organized into themes that were used to inform interview guide development for the in-depth interviews.

### Qualitative interviews

A qualitative descriptive approach was used to inform the qualitative portion of this study [26, 29]. The qualitative descriptive method has been used to capture and describe broad perspectives on health interventions, often as the qualitative component of a mixed-methods study [30]. Qualitative description is a naturalistic approach, appropriate for this study as it recognizes the subjective nature of participants’ preferences. Rather than claiming to advance theoretical or conceptual understanding, our aim is to contribute to quality improvements in future animated, global health communication interventions [26, 30].

Telephone interviews are gaining popularity as a data collection method in qualitative descriptive research, especially as they can support maximum variation purposive sampling and help researchers examine the perspectives of international participants [30].

A majority (*n* = 224, 68%) of survey respondents indicated a willingness to participate in a follow-up in-depth telephone interview. From those who volunteered, maximum variation purposive sampling [31] was used to select 20 respondents from whom we could elicit the widest variety of perspectives (across geographic location, gender, age, years of education, and style rating patterns). Maximum variation sampling has been described as particularly useful in qualitative descriptive research, by Sandelowski and colleagues (2010) [29], as it acknowledges a breadth of diverse perspectives and experiences.

Interview participants were shown short video animations incorporating the animation prototypes they had previously ranked in the online survey. Because the video prototypes were excerpts from real-world MCH interventions, created with collaborating health organizations, the exact health messages varied somewhat across prototypes, including MCH topics such as breastfeeding, early child nutrition and alcohol avoidance in pregnancy. Participants received a single-page reference document containing the eight prototypes and their corresponding names, as presented in the survey. They were then asked to reference the video samples and the prototypes during interviews, which occurred over a two-month period following completion of the online survey. Participants were asked to describe why they felt drawn to or put off by certain prototypes, they were also asked to provide thoughts on characteristics that would be important for globally scalable content.

Interviews were conducted by a co-investigator who is a full-time staff member at Stanford’s Digital Medic South Africa program, a health education and evaluation team based in Cape Town, South Africa. We conducted regular debriefings with the co-investigator who conducted all 20 interviews, as recommended in the literature [32]. This allowed the research team to gain early insights into emerging themes and supported our process of substantive coding. As suggested by McMahon & Winch (2018) [32] debriefings also allowed us to rapidly share emerging data within our production teams and with collaborating stakeholders, thereby shaping the developmental trajectory of ongoing health communication projects.

A combination of conventional content and thematic analysis was used to analyze the interview data, with the intention of keeping the analysis accessible and generating findings that would be readily usable by future creators of animated health communication content. Following each interview, audio-recordings were uploaded and transcribed. Through inductive category development, interviews were coded by two co-investigators (RPC and MA). After reading through the transcripts for the preliminary general analysis, the transcripts were read a second time, with the goal of identifying and highlighting words and phrases that captured key concepts. The co-investigators then made notes of their initial thoughts and impressions with the goal of developing a set of preliminary codes. Specific quotations relevant to each code were collected and organized in a shared Google Drive document. In this way, the research team identified and documented the emerging set of codes, then grouped these into themes, relevant to the development of animated health communication videos for global learners. Rather than assess or strive for high inter-rater reliability that would allow each co-investigator to code entirely independently, we used a process involving constant comparative analysis to independently identify, then verify emerging codes and then organize these into themes. Comparing notes revealed that similar codes were being identified by both co-investigators and validated efforts to ensure that the emerging codes and themes originated from the data, rather than the investigators endeavoring to fit the data into pre-determined categories.

As themes emerged from the early interviews, the interview guide was adapted to include questions focused on these themes. For the purposes of our study, we defined data saturation, in line with prior research [33], as the point at which we had gathered enough information for the study to be replicable and when further coding was no longer feasible (ie: no new codes emerged).

In order to establish trustworthiness, as described by Lincoln and Guba (1985) [34] we consulted with a qualitative researcher at the Heidelberg Institute of Global Health, who reviewed and provided comments on the overall adequacy of this approach. We also created a Google Drive folder containing all audio recording of the interviews, transcripts, data analysis notes, and analytic memos, to establish credibility. In order to ensure transferability, we used purposive sampling and made detailed contextual notes for each respondent.

## Results

### Surveys

**Fig. 2:**
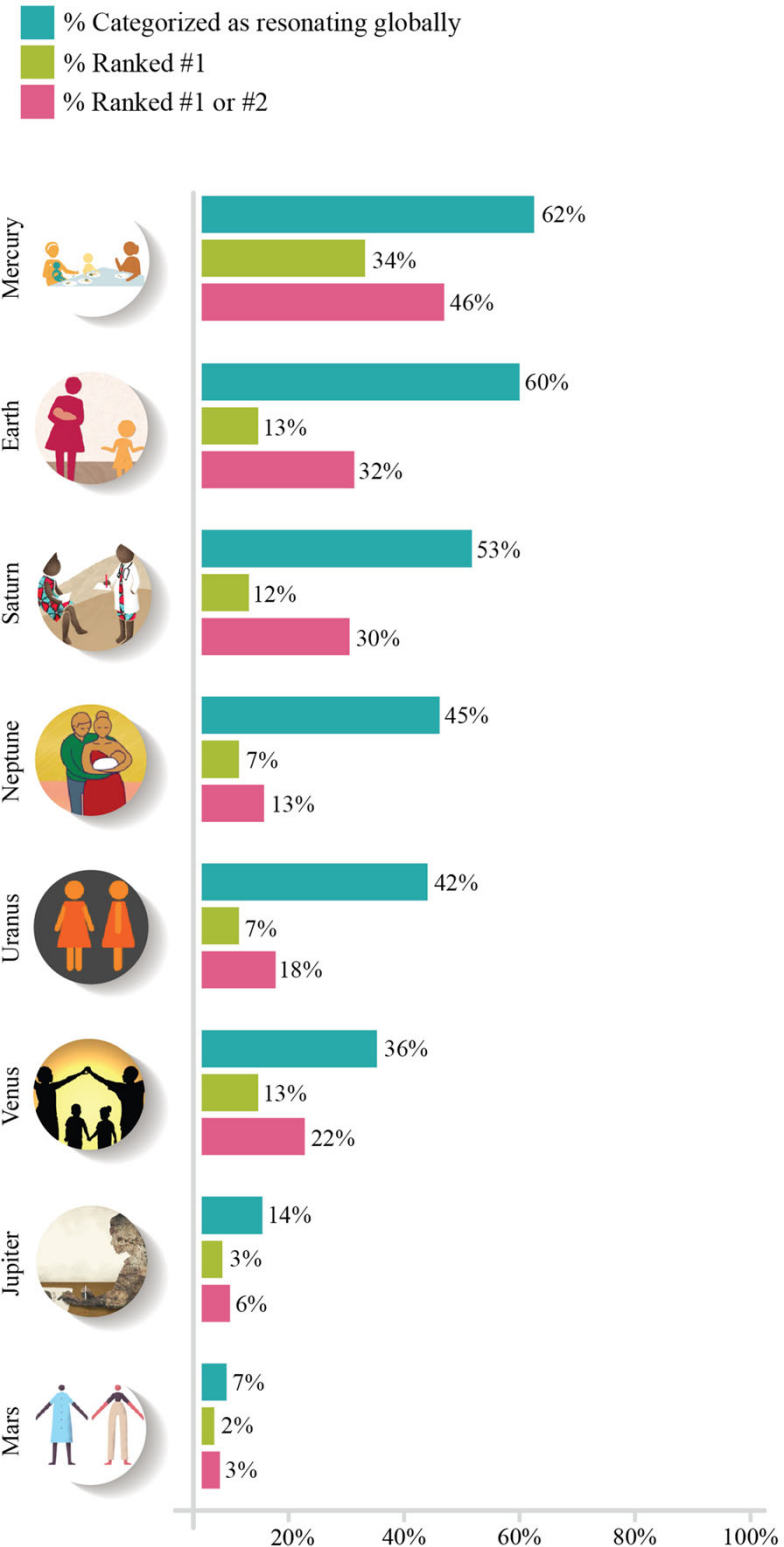
Percentage of respondents categorizing each design prototype by likelihood of global resonance

Most respondents indicated preferences for three or four of the eight design prototypes when asked which of these they felt would be most likely to resonate globally. Across the design prototypes, Mercury was most commonly preferred, with 62% of respondents categorizing it as “would resonate very well for global learners” and 46% ranking it as the first or second most-preferred design prototype. Earth was the second most preferred, with 60% of respondents categorizing it as globally resonant and 32% ranking it as the first or second most-preferred (see Fig. 2).

**Fig. 3:**
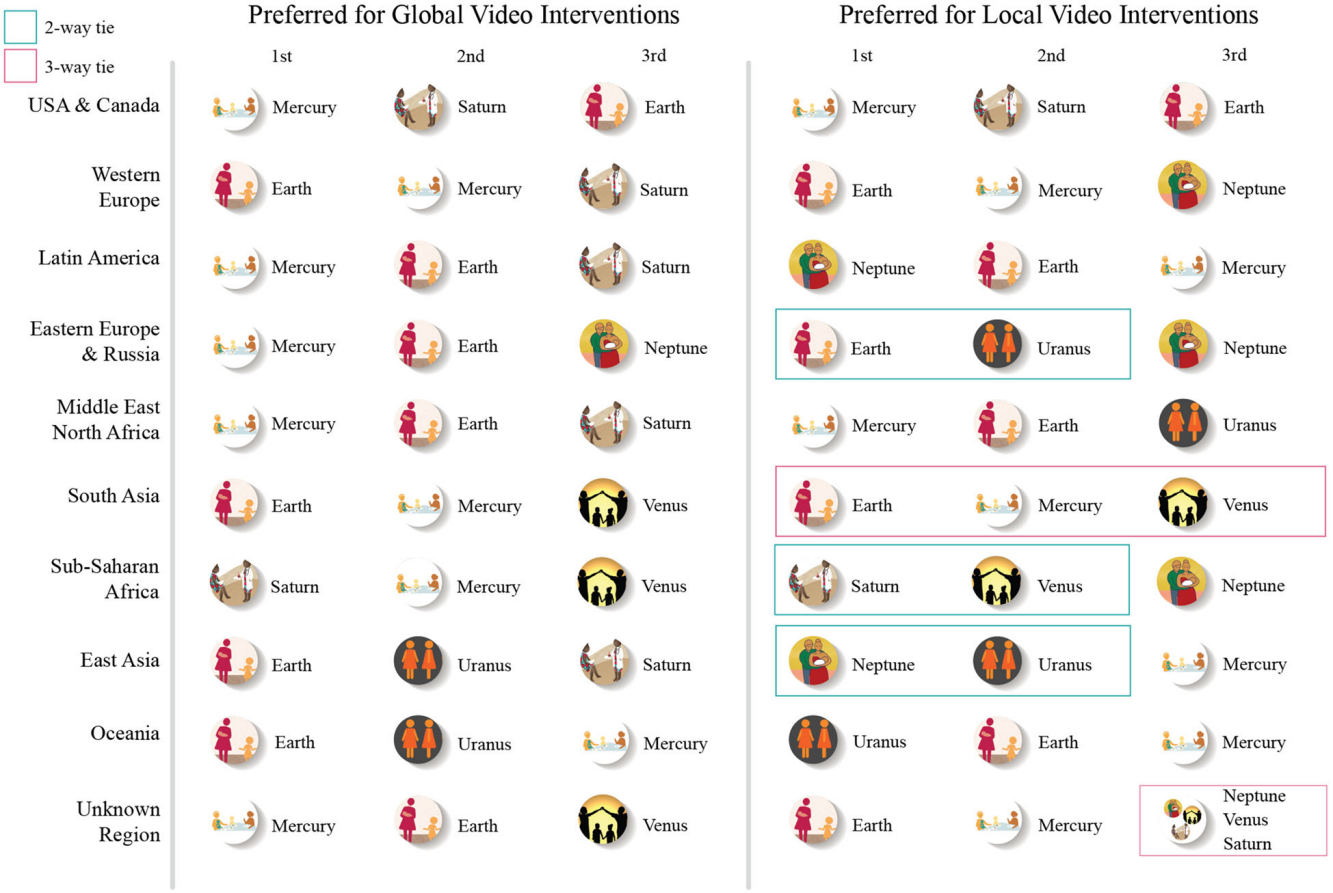
Preference rankings by region of design prototypes expected to resonate globally and locally by respondents

When assessed by region, Mercury and Earth were frequently ranked within the top three most preferred design prototypes. This preference was consistent for both local and global video interventions across respondents who lived in the USA & Canada, Western Europe, Latin America, the Middle East & North Africa, South Asia and Oceania. Respondents living in East Asia did not commonly include Mercury in their top three preference rankings and those in Sub-Saharan Africa tended to rank Saturn higher than Mercury, (see Fig. 3). These results were similar to those found when analyzing data by the World Health Organization’s global regions, a schema that defines fewer (6 rather than 9) global regions [35].

Of note, respondents in some regions indicated different preference rankings locally compared with the design prototypes they expected to resonate globally. For example, in East Asia and Latin America, respondents frequently preferred Neptune, but did not anticipate it to resonate globally.

Similarly, when asked to categorize the design prototypes by how likely respondents felt they would resonate locally and globally, Mercury and Earth were most frequently included (68 and 67%, respectively). Six of the designs (Mercury, Earth, Saturn, Neptune, Venus and Uranus) were included by more than 50%. Respondents were also able to “dislike” designs they felt would not be preferred by local learners in their regions. Venus was disliked by 34% of respondents. Both Jupiter and Mars were disliked by more than 50% of respondents.

**Fig. 4:**
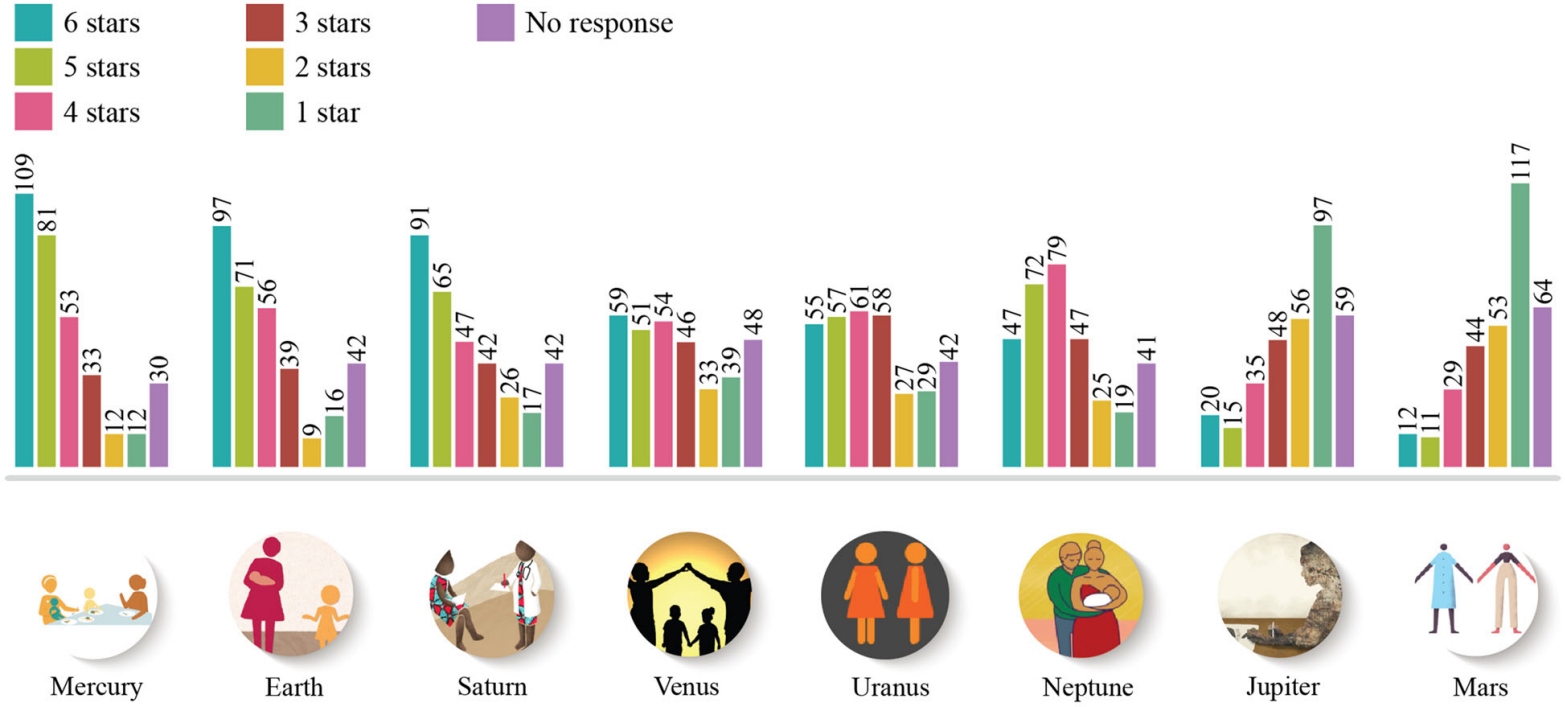
Number of respondents that assigned ratings of 6 stars (best) through 1 star (worst) by design prototype

The star rating exercise produced similar results, with Mercury, Earth and Saturn receiving the highest star ratings, and Mars and Jupiter the lowest. A minority of respondents (14%) opted to provide no response to the star rating exercise (see Fig. 4).

### Interviews

Several questions were consistently explored across all interviews. We asked respondents for their views on a variety of considerations applied to the design of animated content for global audiences. Within this context, respondents were probed regarding visual characteristics that either drew participants to certain characters or repelled them, as well as preferences regarding characters, backgrounds/settings and color choices.

Probing questions posed in the original structured interview guide elicited several themes that provided a more nuanced understanding of the design prototype preferences captured through the survey feedback. During the open coding process, several new themes emerged, including codes that related to the use of a narrative approach to animated video health communication and qualities that make a narrated voiceover credible and engaging. Additional emerging themes included optimal video length, ideal number of learning objectives, as well as the portrayal of family roles in health communication videos. After noting these emerging themes, we adapted the interview guide to specifically probe on these topic areas. In this way, the interview guide remained a living document, responsive to feedback from previous interviews. The investigators agreed that saturation had been reached when interviews 15–20 revealed no new codes, in line with definitions in the literature on qualitative in-depth interviews [36, 37]. Summaries of codes and themes that emerged from the data are presented in Table 2.

**Table 2:** Summaries of main emerging themes elicited during in-depth interviews

Emerging themes	Summary of coded responses
Glocalization	The majority of respondents, in both interviews and open-ended survey comments, expressed that the simplification of character and setting representations was acceptable to them and an effective strategy for designing visuals that would resonate cross-culturally, (ie: Respondent from North America: “Characters with no faces is helpful. Then you don’t take time trying to figure out what they look like.”) Several respondents suggested that simpler visual representations of characters facilitated the individual viewer’s projection of familiar identifiers onto the characters, (ie: Respondent from Latin America: “It’s better if you don’t relate any skin color so that anyone can feel related … When the people were in colors - like blue - and the ones that were in simple shapes, that was very universal. I think Mercury is the best.”) A few respondents felt that extremely simple character representations might not be able to elicit the level of identification needed to transport the viewer in a way that facilitates audience engagement. Most respondents felt that the use of compelling voiceovers and narratives could help to overcome the challenges associated with simple, universally applicable imagery, (ie: Respondent from the North America: “Images should be as ‘neutral’ and universal as possible, but the voiceover and the webpage… should be specific to the target audience.”) In China, one respondent noted that videos reflecting obviously foreign characters and settings might elicit positive, aspirational sentiments, compared with those that are universal or familiar to the viewer. This hypothesis was not echoed by respondents in other countries. In both in-depth interviews and open-ended survey feedback, the prototypes that incorporated ethnic identifiers were more likely to be preferred within regions where those identifiers were felt to be familiar, but less preferred for global resonance, (ie: Respondent from Eastern Europe: “I work with international people… but I remember some African pictures in [the survey]. I loved them but I don’t know how others might take that. Perhaps more universal pictures are better for everyone.” Respondent from Egypt: “these parents don’t look like ours so it’s not for my country.”)
Color	In general, respondents expressed a preference for high contrast or bright, solid colors as a means of supporting viewer engagement, (ie: Respondent from SE Asia: “Some of them appeared to be gloomy and some of them appeared to be vibrant to me. The grey colors were feeling a little dull.”) Bright colors were also suggested way of making content accessible to those in need of visual accommodations, (ie: Respondent from Western Europe: “I prefer high contrast colors because these can also help to account for various disabilities.”) Respondents from different regions mentioned region-specific associations with select colors that should be taken into account when deciding on a universal color palate, (ie: Respondent from SE Asia: “Red is associated with death in Korea.”)
Family portrayals	Several respondents reflected on the importance of considering the potential variation in portrayals of “typical family” structures and depiction of traditional roles. This was specifically referenced with regard to gender norms regarding child caregiving roles. Respondents in both the interviews and qualitative feedback sections of the survey expressed a preference for inclusive representations of diverse family structures, (ie: Respondent from South SE Asia: “It should reflect the different family roles in different places. Grandparents do the bulk of child-rearing in South Korea, so family dynamics might look different….Men are more open to messages that they should invest more time and energy in parenting.”)
Voice	In general, respondents felt voiceovers should ideally be tailored to align closely with both local language and familiar-sounding voices in order to optimally enhance learner identification and engagement with the content. A minority of respondents expressed a fascination with the novelty of an unfamiliar accent but qualified this by noting that they felt many people would stop listening or dismiss messages delivered by a narrator with an unfamiliar accent. Many respondents felt that featuring a child’s voice within the narration was compelling, (ie: Respondent from SE Asia: I felt the energy of the child voice was good…. The voice of the child creates a good mood and resonates well because it has a good energy... The child can be the agent of change.) Other perceived benefits, noted by respondents, included: a) the neutrality/innocence of the child’s voice, b) the feeling that a child’s voice was considered “cute” and was therefore potentially less patronizing or directive than instructions coming from an adult. A minority of respondents felt that a child should only narrate videos for other children as a child’s voice would not carry the same authority as an adult’s voice.
Use of narration/stories	A majority of respondents expressed a preference for a narrative approach to instruction vs. a more traditional approach focused on simply relaying information, (ie: Respondent from Eastern Europe: “It’s a little bit boring when it is just a person telling you information.”) These respondents felt that simple animations were effective as supporting visuals for narratives and could more effectively be universalized, while the voice of the narrator should ideally be tailored to the region.
Video length	In general, respondents expressed a preference for short (2-5 min) videos for health communication purposes, with no more than 2–3 key messages per video, (ie: Respondent from North America: “health messaging of this sort… can only be a couple of minutes long with, ideally, 2–3 messages.”)

## Discussion

Continually evolving health communication approaches and technologies present new opportunities for delivering health information to global audiences. This becomes increasingly important during global public health crises, when evidence-based health information needs to be communicated quickly across global regions. The ability to convey evidence-based health messages globally, including to remote or under-resourced communities, reflects a growing potential to address global health disparities in access to health information [5, 38–42]. However, the ultimate success of these innovations in health communication will depend on their capacity to be compelling, accessible and applicable for individuals in different global regions, with widely differing life experiences.

Based on the results of this study, we offer two sets of considerations that may help to inform the development of more versatile interventions. These include:

- *The degree to which visuals need to be localized for each audience:* Our data suggest that global learners may be willing to accept simplified visuals, designed for broad cross-cultural acceptability. This acceptance is enhanced if the content is localized in other ways, such as the use of locally resonating voiceovers. Localizing the audio only can help to offset the relatively cost- and time-intensive process of customizing visuals for multiple audiences. Care may need to be taken to ensure that social and familial roles are represented in ways that are varied enough to be inclusive of the broadest possible audience. This may mean, for example, including a variety of permutations of family structure or taking care not to perpetuate gender stereotypes around childcare or the provision of food for the family. The goal should be to design for the broadest audience. Approaches to glocalization, successfully used by the children’s entertainment-education industry, include the addition of “cut-ins” or short segments of highly localized video content added strategically at specified points in the video to help viewers identify with it [18, 19]. The thoughtful use of color should also be considered, both with regard to the potential significance of certain color choices in different global regions as well as the opportunity for color to be used in a way that facilitates accommodation of learners with differing abilities. Color may also be used to enhance the viewer experience on mobile devices, with contrasting colors being easier to differentiate on smaller screens.
- *The degree to which voice and narrative need to be localized for each audience*: Our data suggest a general acceptance of narrative approaches to health education and communication. These findings echo existing data that support the role of narrative approaches for health messaging [4, 43]. Narratives may be especially appropriate when developed with input from the target audience, using a human-centered design approach [14], and when accompanied by visuals that invite generalizations and facilitate the creation of analogies by the learner [44]. The use of narratives may also be a powerful way to support the inclusion of audiences with limited literacy and those with rich oral traditions. Storytelling lies at the foundations of the human experience and decades of research underscore its important role in education and healthcare [45]. The characteristics of the narrator need also be carefully considered and may vary by audience and content area. For example, harnessing the power of the child’s voice, especially for relevant topics like maternal-child health and nutrition, could be an innovative way of increasing engagement and overcome reactance towards behavior change messages. Larger-scale studies are needed to further explore this approach.

In this study, our main limitation was the fact that the platform, from which participants were recruited, does not reflect the breadth of socio-economic and educational diversity we ultimately hope to reach with our health communication interventions. Eighty percent of our study participants were female, 52% had more than 18 years of education and almost half of our sample represented ‘western’ culture, residing Canada, the US or Western Europe. Coursera also attracts a majority of users with at least some English language fluency, although the course from which participants were recruited is offered in 5 languages. The users of an online learning platform like Coursera likely have a level of technology savviness and an openness to online learning that could influence their preferences for animation design prototypes. While our participant pool did afford us access to a global pool of culturally diverse learners, it is possible that our results are not representative of people around the world who would engage with video-based health communication.

Secondly, because the video prototypes were excerpts from real-world MCH interventions, created with collaborating health organizations, the exact health messages, while all relating to MCH, were not standardized across prototypes. This could have influenced participants’ preferences for the different animation styles.

Finally, the use of a qualitative descriptive approach can raise concerns that the choice of this method was based on expedience rather than appropriateness and, furthermore, that content and thematic analyses are the easiest, rather than the best, approaches to analyzing the data gathered. In this study, the rationale for this approach, lies in our primary aim of keeping the data closely aligned with the responses of the participants and readily accessible to animated health communication content producers. Given a clear rationale, such as the one we describe here, Vasimoradi et al. (2013) [46] suggest that the qualitative descriptive method may well be the best choice for generating high quality findings that are aligned with the original aims of the study. Especially when interviewing participants for whom English is a second language, a degree of clarity of their qualitative feedback may be lost in overly theoretical or conceptual interpretations of the data.

With these limitations in mind, it is worth noting that while this manuscript was being prepared, the novel coronavirus disease pandemic 2019 led the World Health Organization to declare a global health emergency. We used data gathered during this study to inform the rapid development of an animated, global health communication video that could be used worldwide without adaptation. While we were not able to use one of the exact prototypes featured in this study, due to lack of availability of the artist who created the preferred prototypes, we did apply the principles presented here (ie: simplified visual representations of characters and settings, the use of bright, solid colors and the choice of a short, narrative video with a few key messages). Because the COVID-19 video was intended for immediate global dissemination, without time for translations of voiceover, we were not able to use language in this video and thus, could not apply the findings presented here that relate to voiceovers. Within 10 days, the resulting video [47] was viewed more than 1 million times on various social media platforms around the world (see Additional file 3).


**Additional file 3.** COVID-19 Together We are Stronger (a short, animated wordless video, informed by the findings of this study, ie: featuring characters free of cultural identifiers. The video was used to convey urgent public health messages globally at the start of the COVID-19 pandemic).

## Conclusions

The findings of this mixed-methods study suggest that using simplified animation prototypes, free of obvious cultural identifiers, may increase the acceptability of video-based health communication interventions across global regions. Cultural identifiers include visual details like hairstyles, facial features or traditional dress. Avoiding these characteristics in favor of more universal, icon-style representations of human beings, may help health messages to resonate more broadly across globally audiences. Broad resonance of the visual elements of animated videos could help to scale global health messages more quickly and cost-effectively if these elements are repurposed for different global regions.

Localization of health communication interventions can be achieved through the use of locally resonating narratives and familiar sounding voiceovers. Our findings suggest that learners within a given global region may be more willing to accept simple character representations when the health messages are delivered by a voice actor or narrator whose language and accent are familiar sounding to the learners. In this way, generic-looking characters can adopt a local feel, thereby achieving some degree of “regional targeting” of health messages. Additionally, the use of a narrative approach to presenting health messages, can support the localization of global health messages.

Finally, portrayal of diverse family structures and gender roles can convey a general inclusiveness and acceptance of diversity, which many global learners perceive as an asset to the health messages presented. Given the diverse experiences of global learners, inclusive portrayals of families and the roles of each member within a family, may be an asset when global resonance is the end goal.

By increasing the accessibility of evidence-based health recommendations, through the design of effective, quickly scalable health communication videos, we can begin to meet the needs of a diverse global community. The associated social responsibility for providing equitable global access to these tools demands thoughtful, theory-driven, and evidence-based communication approaches – ones that will engage and empower people from different global regions and backgrounds [11]. Designing health communication interventions, from the outset, with the potential for glocalization may prove to be both an ethical and a practical approach to improving health communication around the world.

## Supplementary Information

**Additional file 2.** Interview Guide (used for the in-depth interviews in this study).

## Data Availability

The survey data that support the findings of this study are available from the corresponding author. All video interventions cited in this study are publicly available and can be accessed through the Digital Medic YouTube channel or by contacting the corresponding author.

## Abbreviations

HCD: Human-centered design
MCH: Maternal child health
