# Supplementary material for: Design preferences for global scale: a mixed-methods study of “glocalization” of an animated, video-based health communication intervention
Source: BMC Public Health. 2021 Jun 25;21:1223. doi: 10.1186/s12889-021-11043-w (PMC8226350; doi:10.1186/s12889-021-11043-w)

Interview tool for Qualitative Universal Usability Study

Introduction:

Thank you for making time to speak with me today. The purpose of this conversation is to understand your preferences of visual styles that we are considering using for our global health education videos.

We are speaking with a number of international learners who have kindly volunteered their time to help us better understand what kind of content is appealing and acceptable to people around the world. Many of the people who we are making this content for live in parts of the world where data costs are high, so the content needs to be visually simple and the file sizes kept small. Our goal is to achieve a style of content that is enjoyable to watch and helps people who want to learn more about their health and the health of their families.

By gathering feedback from you and others in different parts of the world, we will learn more about how we can most effectively engage international audiences.

We really want to learn and improve as a result of this exploration, so please answer candidly and don’t feel you need to sugarcoat anything! Remember, there are no right or wrong answers.

With your permission, I will be recording this interview so that we can transcribe it later. The recording itself will not be used for any other purposes, it will be stored securely on a password protected, encrypted computer, and the recording will be deleted after it has been transcribed.

You may also choose to stop answering my questions at any time and we will end the interview. This should not take more than 45 minutes. Thank you again for your time.

Questions:

1. We are aiming to capture perspectives from a variety of professions, cultures, religions, countries, and languages. Can you tell me about yourself so we can better understand where you are coming from?
   *Probe as needed:*
   1. In which countries have you lived?
   2. Have you lived in urban or rural areas?
   3. What languages do you speak?
      1. Which would you describe as your native language or languages?
   4. What religious groups and cultural groups would you say you are very familiar with?
   5. What is your educational background? Your education goals?
   6. What is your work?
   7. Can you describe your interaction with children?
2. What have your prior experiences been of watching educational videos?
   1. What were the formats of these videos?
   2. Do you have any experience with illustrated or animated videos?
3. What do you think are important considerations when designing animated content for use in many different countries?
   1. Characters
   2. Backgrounds/settings
   3. Colors used, if relevant
   4. Using stories to teach vs simply presenting information
4. Which of the styles presented in the survey do you feel would be most likely to resonate globally? Why did you choose these styles?
   1. Probe regarding character designs
   2. Probe regarding colors and textures
   3. Probe regarding cultural identifiers
   4. Probe regarding portrayals of family and caregiver roles
   5. Probe regarding sound (voiceover, music, sound effects)
   6. Probe regarding length of videos/number of learning objectives
5. Which of the styles do you feel would be less likely to resonate globally?
   1. Why did these styles fail to appeal to you?
   2. What was it about these visual styles that make you feel they would not resonate globally?
6. Please listen to these voiceover samples. *Play 40-60s from these videos: (*<https://www.youtube.com/watch?v=YoEebMFxch8&feature=youtu.be>,

<https://youtu.be/Eddb4_rI1Fw>)

What are your impressions of the voices we have chosen?

- 1. Probe about strengths/pros of using these voices.
  2. Probe about weaknesses/cons of using these voices.
  3. In general what are some characteristics of voices you prefer for this kind of video content?

1. Is there anything I haven’t asked you that you would like to share?

Thank you so much for making the time to speak with me.

1. Thumbnails for recall of style names. (Please send to respondents prior to interview.)


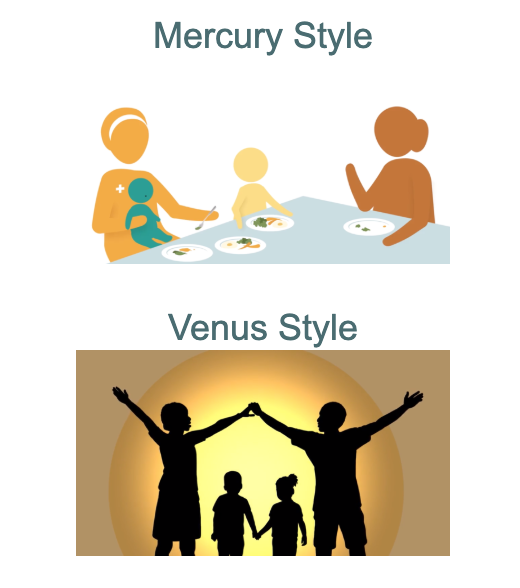

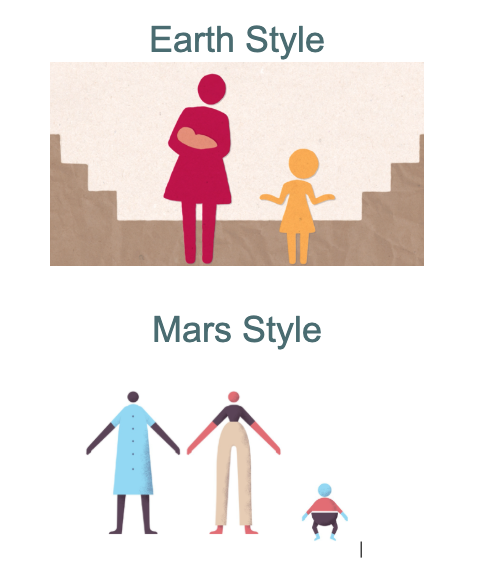


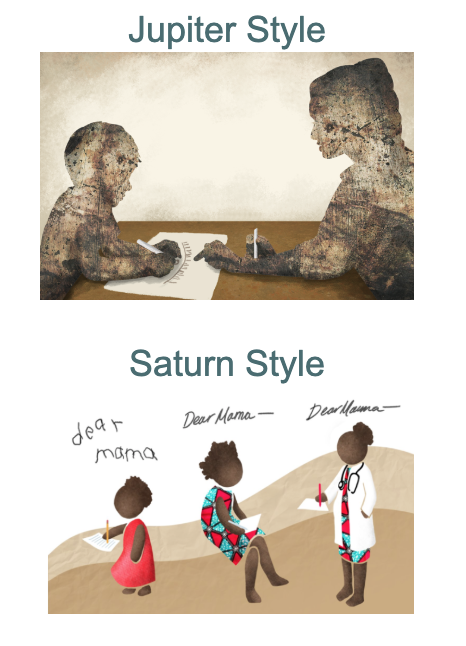

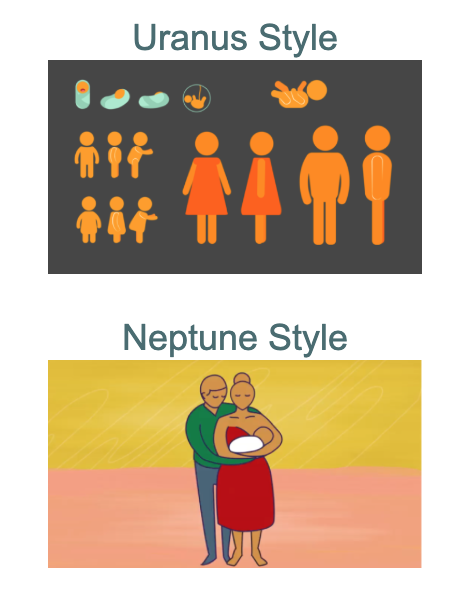

Supplement: Supplementary file 3 — Additional file 2. Interview Guide (used for the in-depth interviews in this study). [file 12889_2021_11043_MOESM2_ESM.docx]
